# Is It Possible to Shift from Down to Top Rank? A Focus on the Mesolimbic Dopaminergic System and Cocaine Abuse

**DOI:** 10.3390/biomedicines9080877

**Published:** 2021-07-23

**Authors:** Inês M. Amaral, Alex Hofer, Rana El Rawas

**Affiliations:** Division of Psychiatry I, Department of Psychiatry, Psychotherapy and Psychosomatics, Medical University Innsbruck, 6020 Innsbruck, Austria; a.hofer@i-med.ac.at (A.H.); rana.el-rawas@i-med.ac.at (R.E.R.)

**Keywords:** drugs of abuse, social interaction, social rank, mesolimbic system, dopamine, social reorganization, animal models, resilience, vulnerability, relapse

## Abstract

Impaired social behavior is a common feature of many psychiatric disorders, in particular with substance abuse disorders. Switching the preference of the substance-dependent individual toward social interaction activities remains one of the major challenges in drug dependence therapy. However, social interactions yield to the emergence of social ranking. In this review, we provide an overview of the studies that examined how social status can influence the dopaminergic mesolimbic system and how drug-seeking behavior is affected. Generally, social dominance is associated with an increase in dopamine D_2/3_ receptor binding in the striatum and a reduced behavioral response to drugs of abuse. However, it is not clear whether higher D_2_ receptor availability is a result of increased D_2_ receptor density and/or reduced dopamine release in the striatum. Here, we discuss the possibility of a potential shift from down to top rank via manipulation of the mesolimbic system. Identifying the neurobiology underlying a potential rank switch to a resilient phenotype is of particular interest in order to promote a positive coping behavior toward long-term abstinence from drugs of abuse and a protection against relapse to drugs. Such a shift may contribute to a more successful therapeutic approach to cocaine addiction.

## 1. Introduction

Impaired social behavior is a common feature of many psychiatric disorders including substance abuse disorders, depression, schizophrenia and autism. In the context of substance use disorders, disturbances in social interaction are associated with an increased drug consumption and challenge treatment success in recovering addicts [1]. Therefore, switching the behavior of the substance-dependent individual toward social interaction activities remains one of the major challenges in drug dependence therapy [2].

However, social interaction between at least two individuals, usually of the same species, yields to the emergence of social ranking in the group. A group is defined as a set of conspecific individuals that remain together and interact with each other more than with other conspecifics [3]. Social rank in a group of individuals constantly monitors one’s standing in relation to others and uses that information to guide behavior [4], such as access to resources and defending territories. Therefore, hierarchical rank has broad effects on physical and mental health due to the risk factors associated with living in a particular rank [5].

How does social rank impact drug taking? How is it believed to be mediated? These questions have been addressed by several research groups (see reviews [6,7,8,9]). However, the conclusions did not emerge in the same direction. In this review, we will overview these studies and address the possibility of switching the profile of a susceptible subordinate individual into a resilient dominant one.

## 2. Social Rank and Drugs of Abuse

Studies in humans [10] and animals [11,12,13] demonstrate that social status is an important determinant of drug taking. Vulnerability to psychostimulants appears to be a feature of socially subordinate and socially stressed animals [14,15]. Indeed, social defeat stress in rats enhanced the acquisition of cocaine self-administration and increased the motivation to seek and take a drug [11,16]. Social defeat is experimentally initiated when a male rodent is introduced into the home cage of an older, aggressive, dominant male [17], triggering depression-like behaviors [18].

Consistent with these findings, socially subordinate male cynomolgus monkeys have been shown to self-administer more total cocaine [13] and to be more sensitive to the relative reinforcing strength of cocaine than dominant monkeys [19]. However, in male rats, social dominance was found to be associated with higher rates of intravenous cocaine self-administration [14]. Next to obvious species differences, the discrepancy in the method used to assess social dominance between non-human primates and rats in the latter study could explain the diverging results [14]. Indeed, in non-human primates, social dominance was identified to be based mainly on aggressive dyadic encounters, while in rats the social rank was assessed based on resource competition for a highly palatable liquid.

Identification of social rank is also possible through the performance of a tube test, based on confrontations within a plastic tube between each possible pairs of a group. A mouse that forces a conspecific out of its way is designated as the ‘winner’, or the dominant. The one that retreats backwards from the tube first is designated as the ‘loser’ or the subordinate [20]. In a study by [21], the submissive mice from four genetically identical male mice, determined by a tube test and the sharing of beneficial resources, had a significantly higher locomotor sensitization in response to repeated injections of cocaine in comparison to the dominant mice. This difference was maintained on the challenge day, thereby suggesting that dominant mice have a weaker behavioral response to cocaine.

In line with the previous studies linking social dominance with resilience to drugs of abuse, it was shown that stress-vulnerable submissive mice exposed to chronic mild stress displayed increased cocaine conditioned place preference (CPP), whereas stress-resilient dominant mice did not differ in preference from their non-stressed state [22]. These results suggest that social submission is associated with vulnerability to stress-induced increases of cocaine preference.

**Figure 1:**
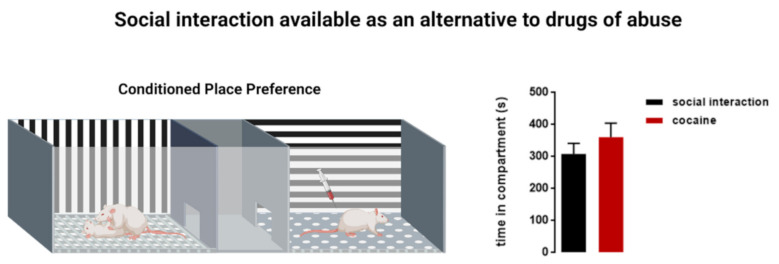
Social interaction as an alternative to drugs of abuse. A conditioned place preference (CPP) apparatus comprises a neutral central compartment connected to two chambers that are different in their context (vertical versus horizontal stripes on the walls) and texture (round versus square holes on the floor). During the conditioning phase, the animal is placed in one chamber of the CPP (in this example, the horizontal compartment) and receives a stimulus such as a cocaine intraperitoneal injection. The animal will then associate the effect of the stimulus with the corresponding context. In a concurrent CPP procedure, another stimulus, such as social interaction with an age and weight-matched conspecific, is made available in the alternative chamber (in this example, the vertical compartment) during the conditioning sessions. Thus, the animals are conditioned with cocaine in one compartment of the CPP and in the opposite compartment, they are conditioned with a social stimulus to incite social play behavior. That way, an animal will associate the context of the vertical compartment with the rewarding effects of social play. On the test day, when the doors to the stimulus-associated compartments are open and the animal is given the choice of where to spend more time, it will spend a similar amount of time in the cocaine-associated, and in the social interaction-associated, compartment. Social interaction reward and cocaine reward have an equal reward value [25,26]. Right panel: time rats subjected to a concurrent CPP protocol spent in each compartment during the test. *n* = 8, Unpaired T-test, t(14) = 0.9666, *p* = 0.3501 (n.s) [26].

In rodents, social interaction was found to be rewarding [23]. When available as an alternative to drugs, dyadic social interaction reduced cocaine preference [24,25,26,27,28] (Figure 1) and prevented cocaine-induced reinstatement of cocaine preference [24]. Interestingly, the dyadic social interaction episodes did not result in the development of an observable hierarchy or a dominance/subordination rank [29,30]. Social hierarchy was determined from the observation of video recordings of the last social interaction conditioning session for signs of dominance/subordination between the pairs. A scoring system was used: h3 hierarchy score for aggressive dominance, defined as three consecutive attacks, such as aggressive grooming, biting, and chasing, by one mouse toward the other; h2 hierarchy score or passive dominance, defined as consistent threatening displacement by one mouse toward the other; h1 hierarchy score, defined as equal status between the pair; and h0 hierarchy score for subordinate behavior, defined as a retreat or withdrawal by one mouse as a reaction to the aggressiveness of the social partner [29]. In the vast majority of social pairs analyzed (i.e., 49 of the total 52 pairs), no visible signs of hierarchy emerged after four episodes of dyadic social interaction (hierarchy score h1) [29,30]. The lack of hierarchical organization in this paradigm was not surprising. Indeed, it seems that just four 15 min episodes of dyadic social interaction with an age and weight-matched conspecific were not enough to induce the establishment of social rank. However, this form of social interaction reward, when available as an alternative to drug use, was suggested as an approach to contracting negative stress effects and increasing resilience to drug use [15].

These findings mostly correlate the state of social dominance, or even the state of equal hierarchy occurring in dyadic social interaction [29,30] to a reduced behavioral response to abused drugs.

Notably, not only hierarchical relations (top-down or down-top), but also peer-to-peer relationships, or the so-called individual network, affect one’s response to drugs of abuse. Compelling evidence demonstrates that peer pressure or social interaction in the same context as drugs can facilitate drug use among adolescents [15,31]. In rats, cocaine self-administration is facilitated in socially housed rats if both animals of a pair have access to cocaine, whereas it is inhibited if only one member of the pair has access to cocaine [32]. Given that rats can behaviorally discriminate between drug-associated and non-drug-associated conspecifics [33], they express a social preference toward a partner with a shared history of drug exposure [34,35]. It appears that the rewarding effects of drugs and dyadic social interaction are enhanced when combined with each other [36,37,38]. However, we focus on dyadic social interaction available in an alternative context to drugs. This form of interaction with an age- and weight-matched conspecific was shown to consistently abolish the preference to drugs [24,25,26], thereby allowing effective coping to build resilience against drug abuse [15].

## 3. Social Rank and the Mesolimbic Dopamine System

The mesolimbic dopaminergic system was identified as the key component in reward assessment [39]. This system is comprised of dopaminergic (DA) neurons with cell bodies in the ventral tegmental area (VTA), which project to forebrain areas including limbic structures such as the nucleus accumbens (NAc), the amygdala and cortical areas including the prefrontal cortex (PFC). Natural rewards, as well as most substances that are abused by humans, including cocaine, increase extracellular concentrations of mesolimbic DA [40,41,42]. Stimulants such as cocaine increase synaptic dopaminergic concentrations by blocking the presynaptic dopamine transporter (DAT), thereby preventing DA re-uptake and increasing DA concentration in the synapse [43].

There are five DA receptors, designated D1 to D5. Based on their structural and pharmacological properties, a general subdivision into two groups has been made: the D1-like receptors, which stimulate intracellular cAMP levels, comprising D1 and D5, and the D2-like receptors, which inhibit intracellular cAMP levels, comprising D2, D3, and D4 receptors [44].

Mesolimbic dopaminergic transmission was suggested as a potential substrate for social ranking. Indeed, in a human study, a positive correlation was seen between dopamine D_2/3_ receptor binding potential in the striatum and social status [45]. Furthermore, using positron emission tomography (PET) imaging to study brain dopaminergic function in individually and socially housed cynomolgus macaques, it was found that social housing increased the amount or availability of DA D_2_ receptors in the basal ganglia of dominant monkeys, and produced no change in subordinate monkeys [13].

In line with these findings, D_2/3_ receptor binding in the NAc shell and dorsal striatum of dominant rats was increased when compared to subordinate rats, and was accompanied by reduced DA levels in the NAc shell [14]. Interestingly, lower concentrations of DA were also found in the NAc of animals living in an enriched environment when compared to individually housed ones [46] but not to group-housed animals [47,48]. Therefore, it seems that the alterations in the mesolimbic system between rats living in enriched versus isolated environments mirror the differences observed in dominant versus subordinate animals, respectively [6,49]. Enriched environments are commonly defined as “a combination of complex inanimate and social stimulation” that usually consists of larger housing cages in a social environment, with a running wheel and toys from different textures changed on a regular basis to facilitate sensory, cognitive and motor stimulation [50,51,52].

Generally, in male monkeys, high D_2/3_ receptors and low DAT availability should lead to less vulnerability to cocaine reinforcement [53]. Similar observations were reported in rodent studies demonstrating the influence of environmental enrichment on dopaminergic function [6,7,54]. In contrast, high D_2/3_ receptor availability and elevated DAT measures were found in the NAc shell of socially dominant rats that maintained higher rates of cocaine self-administration [14]. In subordinate female monkeys, lower D_2/3_ receptor availability as compared to the dominants [53,55], and reduced DAT availability, were correlated to less vulnerability to cocaine reinforcement [53]. Despite all dominant animals sharing the same feature of increased D_2/3_ receptor binding in the striatum [13,14,53], studies on dominant rats [14] and subordinate female monkeys [53] show a direct correlation between DAT levels in the striatum and cocaine reinforcement—relationship that seems plausible as DAT is the main target of cocaine [54,56]. However, mice lacking DAT still self-administer cocaine [57]. It is also possible that changes in D_2/3_ receptors alone may not be sufficient to alter sensitivity to cocaine reinforcement [53]. Indeed, it was shown that the D_2_ receptor is not necessary for cocaine self-administration, but reduced D_2_ receptor levels appear to be involved in the escalation of cocaine intake that contributes to the development of cocaine addiction [58]. Importantly, mice lacking D_3_ receptors displayed an increase in vulnerability to cocaine manifested as enhanced cocaine self-administration and enhanced motivation for cocaine-taking and cocaine-seeking behavior [59].

In the search for biological markers associated with addictive traits, low striatal D_2/3_ receptor availability was linked to high impulsivity [60,61,62] and was predictive of high rates of intravenous cocaine self-administration [60]. Likewise, high DAT expression was found to be associated with impulsive choice [63], characterized by a marked shift of demand from the large and delayed toward the small and soon reward [64]. Indeed, intra-NAc DAT overexpressing rats showed increased impulsivity [64].

Highly impulsive rhesus monkeys have been shown to occupy lower positions in the social dominance hierarchy [65]. Interestingly, it was reported that dominant monkeys showing high D_2/3_ receptor availability were slower to touch a novel object placed in the home cage, consistent with them being less reactive to novelty and less vulnerable to cocaine abuse than subordinates [66]. Lower D_2/3_ receptor availability subordinate monkeys had faster latencies and displayed higher reactivity to novelty [66], consistent with a potential impulsive behavior [67]. Therefore, a lower D_2/3_ receptor coupled to a high DAT expression should lead to more vulnerability to cocaine reinforcement.

Neuropharmacological approaches also proposed a role for DA signaling in social ranking. Indeed, the administration of the D_2_ receptors antagonist, sulpiride, has been shown to attenuate social dominance when the drug is given to high social class macaques and mice [68]. In addition, the highest ranked individuals exhibited a lower bursting activity of VTA DA neurons [21]. Importantly, differences in VTA dopaminergic neurons firing properties were observed as a reflection of individual behavioral differences when animals were gathered in large environments [69]. Remarkably, high D_2_ receptor availability in the striatum was associated with resilience against the development of addiction [70]. Moreover, high sensation seeking may be linked with increased dopaminergic transmission into the striatum, which is in agreement with reduced protection against addictive behavior that is characteristic of individuals with low D_2/3_ receptor binding potentials [71].

Nevertheless, other studies have suggested that the activation of the dopaminergic projections from the VTA to the NAc and subsequent DA release in the NAc transiently facilitates social dominance through activation of D_1_-, but not D_2_, receptor-signaling [72]. The same group also reported that stimulation of mesolimbic dopaminergic neurons seems to promote dominant behavior [73]. Even though these results may appear to be in direct contrast to others that supported a decrease in DA content in the NAc of dominant individuals, they pointed out the importance of the mesolimbic system as a potential substrate for social ranking. Indeed, the roles of D_1_ receptor function in social hierarchy remains less clear. A human PET study has also reported that subjects with low D_1_ receptor availability in the limbic-striatal regions exhibit higher aggression and socially dominant personalities [74], suggesting that D_1_ and D_2_ receptors may have opposite functions for determining social hierarchy. Low D_1_ signaling is also known to be associated with aggression [75], which may assist in attaining higher social dominance, and consequently higher social class. Consistently, low D_1_ functions facilitated social dominance in rodents housed in social groups [76]. In non-human primate social groups, D_1_ facilitation of social dominance remains less clear [76].

Moreover, mitochondrial function in the NAc was reported to be crucial for social hierarchy establishment [77]. Indeed, subordinate rats exhibited reduced mitochondrial complex I and II proteins and respiratory capacity as well as decreased ATP and increased reactive oxygen species production in the NAc [77]. Whereas the micro-infusion of specific mitochondrial complex I or II inhibitors into the NAc reduced social rank, intra-NAc infusion of a brain energy metabolism enhancer prevented the development of a subordinate status [77]. In mice, the metabolic profile in the NAc appears to be associated to social status. Under basal conditions, the subordinate showed lower levels of energy-related metabolites compared to dominants [78]. These findings suggest that mitochondrial function in the NAc might be a potential biomarker for social status.

## 4. Social Rank and Sociability

Individual differences in social behavior emerged after mice with low genetic diversity were living continuously in large groups [69]. A number of studies have shown that dominant-ranked mice exhibited a higher sociability than their subordinates [5,21,79]. Importantly, differences in sociability between the highest and lowest ranked mice were reported to pre-exist as a trait before gathering the animals into tetrads [21]. These findings suggest that social dominance, or a high rank, may positively influence social motivation.

When a hierarchical social group was exposed to chronic social defeat stress, different results were found. Indeed, one study reported decreased social interactions in both high- and low-ranked mice [5], whereas a second study found that low-ranked mice were more likely to develop social aversion [21]. Based on the hypothesis that high-ranked mice would be the ones challenged by stress-exposure, it was found that dominant mice exhibited strong social avoidance, whereas subordinate mice were not affected [78]. The discrepancies in the findings may be attributed to the intensity of the chronic social defeat stress, the time taken in establishing the hierarchy, and the way the results were analyzed. Apparently, when a longer time of cohabitation was investigated (i.e., up to four months), low-ranked mice were more prone to develop social aversion after exposure to chronic stressful conditions [21].

## 5. Effects of Environmental Factors on Social Rank

Given that environmental factors are essential in the determination of the individual vulnerability to drugs of abuse [15], we sought to search for potential effects of the environment on social ranking.

During the formation of social hierarchies, ranks are not determined exclusively by intrinsic factors such as body size, weight, and individual traits, but rather by a range of interacting individual and environmental factors [5,8]. This is evidenced by substantial differences in the behavioral and neurobiological responses following stress exposure in mice of the same inbred strain undergoing equal husbandry and experimental conditions [80]. Environmental factors can have marked effects on dominance hierarchies.

Indeed, neuropathic pain led to a loss of dominance in formerly dominant male mice, such that the previously subordinate mouse became dominant [81]. Other negative environmental factors, such as repeated maternal separation, resulted in impaired social behaviors and induced a lower rank under group-housing conditions in adult male mice [82]. Stress is also known to affect social ranking, as mice subjected to chronic restraint stress showed a reduction in social dominance [83]. Apparently, if one of two male rats was stressed just before their first encounter, stress potentiated a hierarchy-linked recognition memory between “specific” individuals in the favor of a submissive profile for the stressed animal [84]. Under control conditions, the social rank established through a first interaction and food competition test between two male rats was not maintained when animals were confronted one week later [84]. Likewise, peripheral [85] or intracerebroventricular glucocorticoid injection [86] to emerging subordinate rats facilitated the long-term establishment of subordinate rank. Such negative environmental factors diminish the dominant rank or maintain the submissive behavior once a hierarchical relationship is established.

Conversely, when monkeys’ social ranks were manipulated by placing them into new social groups, previously subordinate monkeys showed significant increases in D_2/3_ receptor availability in the striatum, with the largest increase observed in those that became dominant after reorganization [49]. In parallel, cocaine potency as a reinforcer decreased in the majority of animals [49]. These results indicate that the transition to a dominant rank can result in neurobiological adaptations in the brain DA system that have been associated with a decreased sensitivity to cocaine reinforcement in male monkeys.

## 6. Is It Possible to Shift from Down to Top?

As mentioned previously, exposure to a negative environment incites dropping from a top to a down rank, thereby increasing the vulnerability to cocaine abuse-related effects.

One important question is whether it is possible to shift a subordinate or low-ranked profile to a dominant, high-ranked one. Interestingly, a dorsomedial PFC neural population activation was sufficient to quickly induce dominance behavior in the tube test [87]. In line with studies pointing out a role of glucocorticoids in maintaining established subordinate rank, a selective mutation in mouse glucocorticoid receptor genes in DA-innervated neurons including NAc GABAergic neurons, resulted in a higher social ranking as measured by a significantly higher probability to win in a tube test [21], in decreased motivation to self-administer cocaine, and in a lower VTA DA cell firing [88]. Moreover, a permanent, but not a transient reduction in the activity of VTA DA neurons during dominance assessment in the tube test increased the probability of the individual reaching the highest rank [21], thus suggesting that VTA DA neurons activity reduction is in the favor of top rank establishment [21].

**Figure 2:**
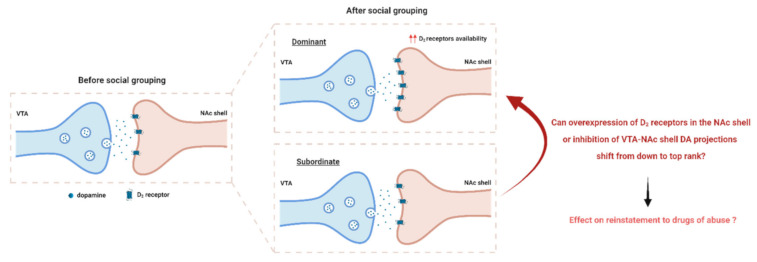
Effect of social grouping on VTA to NAc shell dopaminergic signaling. After social grouping, dominant individuals exhibited higher D_2/3_ receptor availability in the NAc shell [14]. However, D_2/3_ receptor availability was unchanged in subordinates [13]. Higher D_2/3_ receptor binding observed in dominant animals might be due to higher D_2/3_ receptor levels or reduced DA release. Can overexpression of D_2_ receptors in the NAc shell or inhibition of VTA-NAc shell projections shift a subordinate susceptible profile to a dominant resilient profile? What is the subsequent effect of this shift on reinstatement to drugs of abuse? Tackling this question would uncover the cause of higher D2/3 receptor binding observed in dominant animals. VTA, ventral tegmental area; NAc shell, nucleus accumbens shell.

As D_2/3_ receptor binding was elevated in the NAc shell of dominant rats when compared to subordinate rats, and was accompanied by reduced DA levels [14], the question that arises is whether an inhibition of the activation of VTA DA neurons projecting to the NAc shell specifically would be able to switch from a subordinate to a dominant rank and/or whether an overexpression of DA D_2_ receptors in the NAc shell would be sufficient to promote a high rank profile (Figure 2). Manipulations of the VTA to NAc shell projections would uncover the cause of higher D_2/3_ receptor binding observed in dominant animals. Indeed, it is not clear whether higher D_2_ receptor availability is a result of increased D_2_ receptor density or reduced DA release from VTA to NAc neurons [6,53,89,90]. The next question is to determine the subsequent impact of the down-to-top switch on the effects of drugs. Indeed, there is a gap in our understanding of the effects of social ranking, identified based on non-aggressive selections, on the reinstatement of cocaine seeking, particularly stress-induced reinstatement of cocaine seeking [91,92]. This knowledge would help to elucidate why dominant individuals are more resilient to cocaine relapse provoked by stressful stimuli than submissive individuals are.

**Figure 3:**
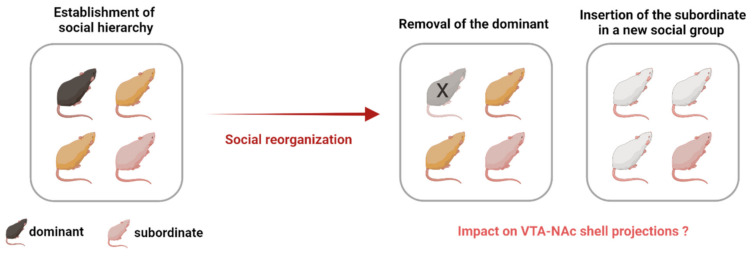
Social reorganization after the establishment of social hierarchy. Social ranks can be manipulated by removing the dominant so that a new dominance can be established, or social reorganization can occur by placing the subordinate into a new social group. What is the impact of social rank reorganization on the mesolimbic system, particularly on the VTA-NAc shell projections? In addition, what is the subsequent impact on the effects of drugs of abuse? Answering these questions will elucidate whether attaining dominance or moving to a new social group can result in adaptations in the VTA-NAc shell projections, possibly affecting the response to drugs of abuse.

The last remaining question is what happens to these VTA to NAc shell projections in the case of social reorganization [49], or in case the social rank in a group changes (Figure 3). A previous study found a substantial increase in D_2/3_ receptor availability in the caudate and putamen nucleus in monkeys who had been subordinate but became dominant when placed in their new social groups [49]. Such a social reorganization in male monkeys was compared to a switch in a relative enriched environmental condition resulting in adaptations in the brain DA systems [49]. Simply changing the social environment appears to be enough to increase D_2/3_ receptor availability, as evidenced by a moderate increase in the caudate nucleus of subordinate monkeys that did not increase their rank in the new social groups [49], thereby supporting the contribution of an environmental alteration to a successful therapeutic approach to cocaine addiction [49]. However, the change in environmental factors should provide the possibility for improvement (rank shift from subordinate to dominant or rank stability as subordinates in a new environment), as depriving mice from an enriched environment was perceived as a stressful and aversive experience that increased vulnerability to cocaine [93].

The modulation of the mesolimbic system can also occur via manipulations of the systems interacting with the mesolimbic DA pathway. A possible candidate is the serotonergic (5-HT) system. The 5-HT system can influence the abuse-related effects of cocaine through its interactions with the DA system [94]. The raphe nuclei, containing the serotonergic cell bodies, projects to key brain areas involved in drug abuse, including the VTA, the NAc and the PFC. Administration of serotonin transporter inhibitors that enhance 5-HT levels can reduce the firing and burst rate of DA cells in the VTA and negatively modulate the abuse-related effects of cocaine [94], consistent with a dominant profile. Several studies have shown that this 5-HT system contributes to the formation of social hierarchy [95]. Indeed, dominant male adult vervet monkeys have approximately twice as high whole-blood serotonin concentration compared to subordinate adult males [96]. Interestingly, the shift from the down to the top position in the social hierarchy was accompanied by an increase in 5-HT levels [95]. After removing the dominant monkey of the group, the administration of drugs that enhance serotonergic activity promoted the acquisition of a dominant profile [97]. However, the administration of drugs that reduce serotonergic function promoted a subordinate profile [97]. Thus, through the manipulation of the 5-HT system, the mesolimbic DA can be modulated and can affect the social rank of the individuals, as well as the abuse-related effects of cocaine.

## 7. Conclusions

Socioeconomic status impacts human health and susceptibility to develop psychiatric disorders such as substance use disorders [98,99]. The social environment has a powerful influence on how individuals experience and cope with negative encounters. Regardless of whether this negative encounter was an adverse environmental condition or an exposure to drugs of abuse, social behavior and social rank in particular, are important contributors to individual differences [100] and to the individual responses to environmental challenges [101]. However, for most of the studies, social behavior and rank have been identified on the basis of a detailed observation of pairs. However, it is necessary to study animals in larger groups, rather than in pairs, and also to track individual differences within the group rather than looking at the group as a whole [102]. Indeed, only a minority (around 15%) of rats may continue to take cocaine, while the large majority may be resilient and readily give up cocaine use in favor of a non-drug alternative [103].

Identifying the neurobiology of a potential rank switch to a resilient phenotype is of particular interest to promote a positive coping behavior toward long-term abstinence from drugs of abuse and a protection against relapse to drugs. Such a switch may contribute to a more successful therapeutic approach to cocaine addiction.

## Data Availability

The data presented in this study should be publicly available and cited in accordance with journal guidelines.
